# The Framework Convention on Tobacco Control (FCTC) and Japanese anti-tobacco measures

**DOI:** 10.1186/1617-9625-4-3

**Published:** 2008-07-31

**Authors:** Kazunari Satomura, Suketaka Iwanaga, Megumi Noami, Ryota Sakamoto, Keiko Kusaka, Takatoshi Nakahara

**Affiliations:** 1 Dept. of Public Health, Faculty of Medicine, Kyoto University, Japan

## Abstract

Japanese anti-tobacco measures are reviewed and checked the relationship between the FCTC and its changes. Japan is making efforts to follow the FCTC, but it is insufficient and present anti-tobacco measures seem to have only a little impact on decreasing smoking rates. More effective measures should be developed for reducing smoking rates and for making smoke-free society.

## Introduction

Smoking rates in Japan are quite high compared to other developed countries. Japan ratified the Framework Convention on Tobacco Control (FCTC) on 8th June, 2004. The FCTC was enacted on 2nd 2005. We checked Japanese anti-tobacco measures from the historical viewpoints and studied necessary measures for the FCTC.

### 1. Regulations before 2004

From 1898 to 1985, the tobacco industry had been monopolized by the government. The tax income from the tobacco industry was very large and used for the war expenditures and so forth. There were few regulations before the end of the Second World War. The remarkable regulation for smoking before the end of the war is The Law of Prohibition of Smoking by Minors in effect since 1900.

This law contains following items;

1) Smoking is prohibited before twenty years of age.

2) Offenders have smoking goods confiscated.

3) A person with parental authority or a supervisor who does not stop smoking by minors is fined.

4) Retailers of tobacco or smoking goods should check the age of the purchasers. If retailers sell tobacco or smoking goods to minors, they are fined.

This law is still ineffect and even after enactment of the FCTC there are few arguments for lowering the age of smoking. After the 1960s, negative health effects of smoking have become well known.

The Ministry of Health and Welfare has set a policy of smoking as follows;

1. Ban of smoking by minors

2. Separation of smoking areas

3. Decrease consumption of tobacco

However, it was difficult to reduce the consumption of tobacco for the government as the tax income was so large (about 20% of all tax income).

Since 1972, warnings have been printed on the packages, but the warning was very mild ("Too much smoking might have negative health effects")

As the negative health effects of smoking came to be known more widely, since 2000 the government started " Healthy Japan 21" which contains anti-smoking tobacco measures. "Healthy Japan 21" is a health promotion framework which will end in 2010 [1]. One of the targets is smoking. To reduce smoking rates, four actions were taken.

Four actions are followings:

1. Diffusing negative health effects of smoking

2. Banning of minors' smoking

3. Prevention from passive smoking

4. Assisting quit smoking

To support " prevention from passive smoking", a new law was enacted in 2003. That is the Health Promotion Law and article 25 in this law is as follows;

Persons in charge of management at facilities used by large numbers of people, such as schools, gymnasiums, hospitals, theaters, viewing stands, assembly halls, exhibition halls, department stores, offices, public facilities, and eating and drinking places shall endeavor to take necessary measures to protect users of these facilities from being exposed to passive smoking (passive smoking refers to being forced to inhale other people's cigarette smoke in an indoor or equivalent environment).

Except laws for prevention of fire by cigarette smoking, the Law of Prohibition of Smoking by Minors and the Health Promotion Law are only laws for smoking regulation. After the end of the monopolization, the Tobacco Institute of Japan was established [2] and voluntary restrains were put in place.

These voluntary restrains include:

1. Advertisements by TV, radio, cinemas, electric bulletin boards and internet will not be performed. Also those by signboard or in public transport will not be done except in smoking places.

2. Advertisement by printing matters for youth will not be performed.

3. Advertisement for youth will not be done.

4. In the advertisement, health warnings, and volumes of nicotine and tar should be shown. No misleading words for tobacco should be used.

5. On the package, health warnings and volumes of nicotine and tar should be printed.

### 2. Regulations after 2004

After the end of the monopolization, activities of the tobacco company [3] is regulated by the Tobacco Industry Law. For the FCTC, this law was revised. By this revision health warnings are printed on the package.

The warnings are as follows;

• "Cigarette smoking is a cause of lung cancer. According to epidemiological studies, smokers are at two to four times greater risk of death from lung cancer compared with non-smokers. (For details, please refer the website of the Ministry of Health, Labour and Welfare: )"

• "Cigarette smoking increases your risk of cerebral stroke. According to epidemiological studies, smokers are at 1.7 times greater risk of death from cerebral stroke compared with non-smokers. (For details, please refer the website of the Ministry of Health, Labour and Welfare: )"

• "Cigarette smoking increases your risk of deteriorating pulmonary emphysema. (For details, please refer the website of the Ministry of Health, Labour and Welfare: )"

• "Cigarette smoking increases your risk of myocardial infarction. According to epidemiological studies, smokers are at 1.7 times greater risk of death from myocardial infarction compared with non-smokers. (For details, please refer the website of the Ministry of Health, Labour and Welfare: )"

• "Cigarette smoking during pregnancy can be one of the causes of fetal developmental disorders and premature delivery. According to epidemiological studies, babies of pregnant women who smoke are at two times greater risk of low birth weight delivery and three times greater risk of premature delivery compared with babies of non-smoking pregnant women. (For details, please refer the web site of the Ministry of Health, Labour and Welfare: )"

• "Cigarette smoking damages the health of the people around you, especially infants, children and elderly persons. Be cautious not to bother the people around when you smoke."

• "Cigarette smoking causes nicotine dependency to various degrees among people."

• "Cigarette smoking in minors causes greater dependency and more harmful health effects. Minors should never be encouraged to smoke."

• "The words, "Lights", "Super Lights", "Ultra Super Lights" or "Mild" on the packages do not mean less harmful health effects compared with other cigarettes"

The warnings are scientifically correct but have little or no impact.

To prevent youth from buying tobacco at the vending machines (in Japan vending machines are everywhere and cigarettes are easy to buy). An ID card system will be introduced in 2008.

### 3. What is necessary for the FCTC in Japan

**Figure 1:**
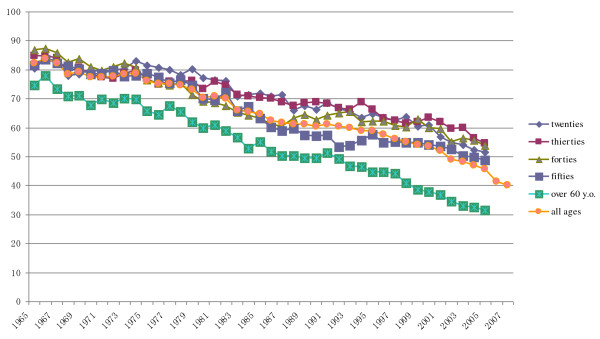
Change of smoking rates in male.

**Figure 2:**
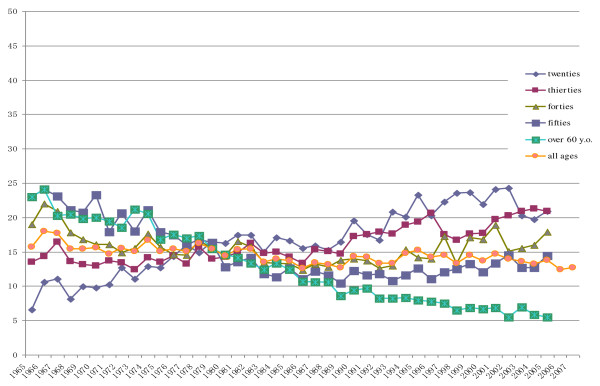
**Change of smoking rates in female**.

**Figure 3:**
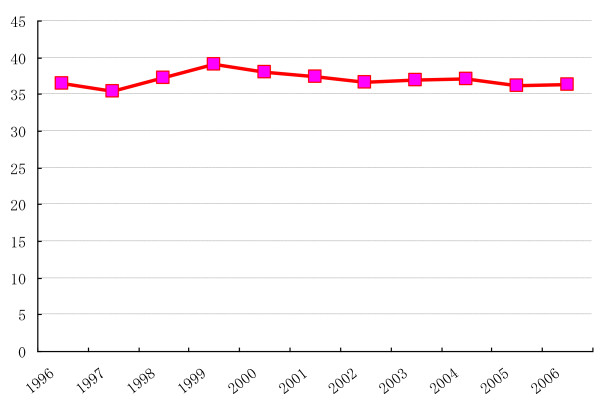
**The Sum of Cigarettes Sales (unit billion US$)**.

Figures 1 and 2 show the historical change of smoking rates and figure 3 shows reduction of cigarettes consumption. The previous anti-smoking measures seem to have had only a small impact on these parameters. In Japan, reducing smoking rates through the use of price controls is not yet accepted and the restrictions on tobacco advertisements are still only voluntary restrains. To improve anti-tobacco measures and decrease smoking rates, raising tobacco prices should be done first of all. Furthermore new measures targeting young female smoking should be developed.

## Competing interests

The authors declare that they have no competing interests.

## Authors' contributions

SI and KK collected data of before the FCTC in Japan and discussed their meaning. MN and RS collected data after the FCTC in Japan and discussed their meaning. TK and KS summarized these discussion and data, also checked these results. KS write this paper according to our results. All authors read and approved the final manuscript.
